# Liouville Integrability in a Four-Dimensional Model of the Visual Cortex

**DOI:** 10.3390/jimaging7120277

**Published:** 2021-12-17

**Authors:** Ivan Galyaev, Alexey Mashtakov

**Affiliations:** 1 V. A. Trapeznikov Institute of Control Sciences of RAS, 117997 Moscow, Russia; ivan.galyaev@yandex.ru; 2 Ailamazyan Program Systems Institute of RAS, 152021 Veskovo, Russia

**Keywords:** model of vision, visual cortex, sub-Riemannian geometry, geodesics, curvature, optimal control problem, integrability

## Abstract

We consider a natural extension of the Petitot–Citti–Sarti model of the primary visual cortex. In the extended model, the curvature of contours is taken into account. The occluded contours are completed via sub-Riemannian geodesics in the four-dimensional space M of positions, orientations, and curvatures. Here, $M=R^{2}\times \mathrm{SO}\left(2\right)\times R$ models the configuration space of neurons of the visual cortex. We study the problem of sub-Riemannian geodesics on *M* via methods of geometric control theory. We prove complete controllability of the system and the existence of optimal controls. By application of the Pontryagin maximum principle, we derive a Hamiltonian system that describes the geodesics. We obtain the explicit parametrization of abnormal extremals. In the normal case, we provide three functionally independent first integrals. Numerical simulations indicate the existence of one more first integral that results in Liouville integrability of the system.

## 1. Introduction

The principles of biological visual systems are of great interest among researchers in many fields of science. An important problem is the development and study of realistic mathematical models describing a certain stage of visual signal processing. In this paper, we perform a study of a mathematical model that describes a mechanism of visual signal processing by the primary visual cortex of the human brain.

Human vision is a complex process that has not yet been fully understood. Perception of visual information starts in the eye. A ray of light hits the retina and causes the activation of light-sensitive receptors. The retina is a part of the brain hierarchically organized in several layers. The first layer consists of light-sensitive receptors. In the next layers of the retina, bipolar and ganglion cells are contained. Here, the primary processing of the visual signal takes place. Furthermore, the visual signal arrives through the optic nerve to the lateral geniculate nucleus (LGN) of the thalamus. Afterward, the signal is transmitted to the visual cortex of the brain.

The mechanism of visual signal processing by neurons of the retina and LGN have been extensively studied. The scale-space theory [1] proposed a mathematical model for this stage. This theory was inspired by the properties of the Gaussian kernel and its derivatives as regularized differential operators as well as solutions to the linear diffusion equation. The receptive fields of bipolar and ganglion cells as well as LGN neurons are well approximated by filter profiles based on the Gaussian kernel and Gaussian derivatives. Their work is modeled by the action of the filter to the input signal. Mathematically, this operation is defined as the convolution of two functions.

After LGN, the signal is transmitted to the visual cortex of the brain. Physiological research indicates that the visual cortex is composed of multiple layers. The research by Hubel and Wiesel [2] has made significant progress in understanding the principles of the primary visual cortex V1. In particular, they found that the receptive fields of V1 neurons are elongated rather than rounded. The cells of V1 are capable of detecting segments of contours with different orientations from the whole image. Mathematically, the work of V1 cells can be understood as a lift of a 2D input image to the extended space of positions and orientations $\mathrm{SE}\left(2\right)=R^{2}\times \mathrm{SO}\left(2\right)$.

The mathematical model of V1 as a sub-Riemannian structure on the Heisenberg group was proposed by J. Petitot [3]. Then, this model was refined by G. Citti and A. Sarti [4] as a sub-Riemannian structure on the Lie group $\mathrm{SE}\left(2\right)=R^{2}\times \mathrm{SO}\left(2\right)$. Here, SE(2) models the configuration space of neurons V1, which can be understood as the space of positions $R^{2}$ and orientations SO(2). According to this model, the process of completion of occluded contours occurs by minimizing the excitation energy of neurons that perceive visual information in the areas of the observed scene that are occluded. This process can be interpreted as the action of the hypoelliptic diffusion operator studied in [5,6,7]. The resulting curves are sub-Riemannian length minimizers in SE(2). Their exact parameterization was obtained in [8]. Such curves are used for image inpainting [9] and for the explanation of some visual illusions [10].

The principles of biological visual systems are actively used in computer vision. Based on these principles, effective methods of image processing are created: enhancement, segmentation, inpainting, and feature detection. For example, in [11], the authors describe an approach that is based on lifting an image into the extended space of positions and orientations. After such lifting, the sub-Riemannian length minimizers are used to detect the salient lines [12,13,14]. This approach is actively used in medical image processing.

**Figure 1:**
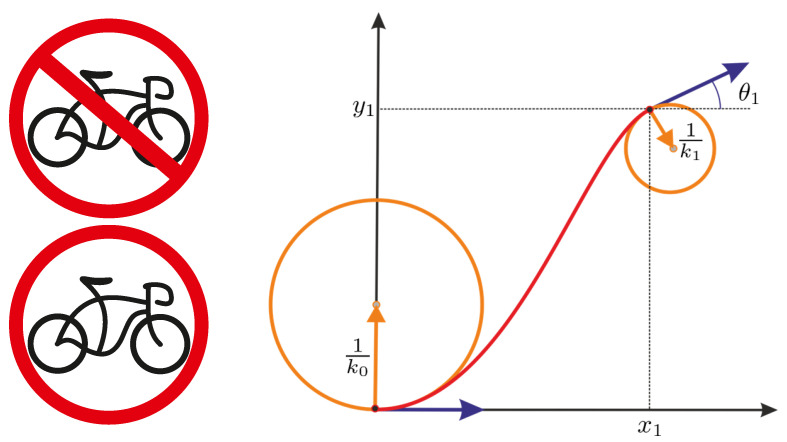
In the four-dimensional model of the visual cortex, an occluded contour is completed via the planar projection of a sub-Riemannian length-minimizer in the space $R^{2}\times \mathrm{SO}\left(2\right)\times R=M∋\left(x,y,\theta ,k\right)$ of positions, orientations, and curvatures. In the left column, we show an example of the image with partially occluded contours and the complete image. In the right column, we show a trajectory that satisfies the given boundary conditions. The curvature is visualized as its reciprocal—the radius of the osculating circle.

The experimental data [15] (see also [16]) suggest that not only detectors of orientation but also detectors of curvature exist in V1. In this paper, we consider the mathematical model of the visual cortex ([17], p. 57) [18] that is obtained by extension of the classical Petitot–Citti–Sarti model. The extension is performed by taking into account the curvature of contours of the observed image. This leads to a sub-Riemannian structure in the four-manifold $M=R^{2}\times \mathrm{SO}\left(2\right)\times R$, where the fourth component means the curvature. In the four-dimensional model of the visual cortex, a partially occluded contour is completed via the planar projection of a sub-Riemannian length-minimizer in *M* that satisfies the boundary conditions (position, orientation, and curvature) obtained from the boundary of the occluded region. See Figure 1.

In [18], the authors show that the 4D model allows for better perceptual grouping and completion of complex images than the classical 3D model. They consider the completion of images via minimal surfaces in the 4D model. In this paper, we study the related problem of finding length-minimizers of the associated sub-Riemannian manifold. In analogy with [9], knowledge of length-minimizers provides a method for the completion of isophotes of corrupted images. Such a method is an alternative to the minimal surface method, proposed in [18], where the linearized Euler–Lagrange equation is used to compute the surface. Such a linearization provides an approximation for the geodesic flow. We expect that the usage of the precise length-minimizers will provide a more accurate method for image completion since it does not involve linearization.

In this article, we consider the problem of sub-Riemannian geodesics in the space *M* of positions, orientations, and curvatures. We formulate the problem as an optimal control problem in Section 2. In Section 3, we prove complete controllability of the system and the existence of optimal controls. Then, in Section 4, we apply a necessary optimality condition—the Pontryagin maximum principle (PMP)—and examine the Hamiltonian system of PMP. We obtain explicit parametrization of abnormal extremals and provide a numerical investigation resulting in the Liouville integrability of the normal Hamiltonian system of PMP. In conclusion, we summarize the main results.

Note that the question of integrability of the geodesic flow in *M* is important both for theory and for applications. Liouville integrability ensures the absence of undesirable chaotic behavior. It guarantees that the trajectories of the system remain close to each other under a small perturbation of the initial value. This property is highly important for the stability of numerical schemes of integration. Note that integrability is a rare phenomenon. In the common case, a system of ODE is not integrable. In [19], the authors show that, even in the simplest case of Carnot groups, the sub-Riemannian geodesic flow is not integrable already in dimension 8 (and consequently in higher dimensions).

## 2. Optimal Control Problem

We consider the following control system:

(1) $$\left\{\begin{array}{c} \dot{x}=u_{1}\cos\theta ,\\ \dot{y}=u_{1}\sin\theta ,\\ \dot{\theta }=u_{1}k,\\ \dot{k}=u_{2}, \end{array}\right.\;\begin{array}{c} (x,y,\theta ,k)=q\in \mathrm{SE}(2)\times R=M,\\ \left(u_{1},u_{2}\right)\in R^{2}. \end{array}$$

For the trajectory corresponding to the control $\left(u_{1}\left(t\right),u_{2}\left(t\right)\right)$, t∈[0,T], T>0, we define a cost functional—the sub-Riemannian length of this trajectory:

(2) $$l=\int_{0}^{T}\sqrt{u_{1}^{2}\left(t\right)+u_{2}^{2}\left(t\right)}\;dt.$$

We study the problem of finding a Lipschitzian curve γ:[0,T]→M—that is a trajectory of system (1), satisfying the given boundary conditions

(3) $$\gamma \left(0\right)=q_{0},\;\gamma \left(T\right)=q_{1},\;q_{i}\in M,$$

and having the minimal sub-Riemannian length l(γ)→min.

**Remark** **1.** *It is easy to check that system* (1) *is invariant under parallel translations and rotations in the plane (x,y). Due to this, without loss of generality, we can reduce the problem for an arbitrary $q_{0}=\left(x_{0},y_{0},\theta_{0},k_{0}\right)$ to the case $q_{0}=\left(0,0,0,k_{0}\right).$*

## 3. Complete Controllability of the System

The first question that arises when studying problems (1)–(3) is the existence of an admissible trajectory connecting boundary conditions (3). If for any $q_{0}$, $q_{1}\in M$ the answer is positive, then the control system is called completely controllable. Let us investigate the controllability of system (1) using the technique of geometric control theory [20].

System (1) has the following form:

(4) $$\dot{\gamma }=u_{1}X_{1}+u_{2}X_{2},$$

where the vector fields near the controls are given by

$$X_{1}=\left(\begin{array}{c} \cos\theta \\ \sin\theta \\ k\\ 0 \end{array}\right),\;X_{2}=\left(\begin{array}{c} 0\\ 0\\ 0\\ 1 \end{array}\right).$$

We investigate the controllability using Chow–Rashevskii theorem. In our case, it suffices to check that the rank condition is satisfied. To do this, we calculate the following Lie brackets of the fields $X_{i}$:

$$X_{3}=\left[X_{1},X_{2}\right]=\left(\begin{array}{c} 0\\ 0\\ -1\\ 0 \end{array}\right),\;X_{4}=\left[X_{1},X_{3}\right]=\left(\begin{array}{c} -\sin\theta \\ \cos\theta \\ 0\\ 0 \end{array}\right).$$

For the matrix composed of the vector fields $X_{1},\ldots ,X_{4}$ we have

(5) $$\det\left(\begin{array}{cccc} \cos\theta & 0 & 0 & -\sin\theta \\ \sin\theta & 0 & 0 & \cos\theta \\ k & 0 & -1 & 0\\ 0 & 1 & 0 & 0 \end{array}\right)\equiv 1.$$

We conclude that the rank of the matrix is four, so the vector fields $X_{i}$ are linearly independent. Therefore, they define a basis of the tangent space $T_{q}M$ at every point *q*. Thus, we see that all of the conditions of the Chow–Rashevskii theorem are satisfied and we obtain the following result.

**Theorem** **1.** *The control system* (1) *is completely controllable.*

**Remark** **2.** *Since the values $u_{i}$ are unbounded, condition* (4) *is equivalent to $\dot{\gamma }\in \Delta_{\gamma }=\mathrm{span}\left(X_{1}\left(\gamma \right),X_{2}\left(\gamma \right)\right),$ where the family of planes* Δ *is called the distribution. Due to condition* (5), *the growth vector of the distribution* Δ *equals (2,3,4). Such systems are called structures of Engel type.*

Furthermore, a question of the existence of optimal trajectories arises: does there always exist an admissible trajectory satisfying conditions (3), on which the functional (2) reaches its minimum value? For our problems (1)–(3), the answer is positive. The existence of optimal trajectories is guaranteed by the Filippov theorem [20,21].

## 4. Pontryagin Maximum Principle

Before proceeding to the examination of extremal trajectories, let us reduce the problem under consideration to a simpler one. By virtue of Cauchy–Schwarz inequality, the original problem is equivalent to the problem of minimizing the action functional

(6) $$J=\int_{0}^{T}\frac{u_{1}^{2}\left(t\right)+u_{2}^{2}\left(t\right)}{2}\;dt\to \min.$$

Apply to problems (1), (3), (6) a necessary condition of optimality—Pontryagin maximum principle (PMP)—we introduce the Pontryagin function:

$$h_{u}^{\nu }=\langle p,\sum_{i=1}^{2}u_{i}X_{i}\rangle +\nu /2\sum_{i=1}^{2}u_{i}^{2},\;p\in T^{*}M,\;\;\nu \leq 0.$$

PMP states that, if (u(t),q(t)), t∈[0,T], is an optimal process, then the following conditions hold:

1. Hamiltonian system $\dot{p}=-\frac{\partial h_{u}^{\nu }}{\partial q},\;\;\dot{q}=\frac{\partial h_{u}^{\nu }}{\partial p}$;
2. Maximum condition $h_{u(t)}^{\nu }\left(p\left(t\right),q\left(t\right)\right)=\max_{u\in R^{2}}\;h_{u}^{\nu }\left(p\left(t\right),q\left(t\right)\right)$;
3. Nontriviality condition (p(t),ν)≠(0,0)∀t∈[0,T].

Denote $h_{i}=\langle p,X_{i}\rangle .$ The Pontryagin function takes the form

$$h_{u}^{\nu }=u_{1}h_{1}+u_{2}h_{2}+\nu /2\left(u_{1}^{2}+u_{2}^{2}\right).$$

In the formulation of PMP, without loss of generality, it suffices to consider two cases: ν=0, the abnormal case, and ν=−1, the normal case. Next, we consider both cases in detail.

### 4.1. Abnormal Case ν=0

The Pontryagin function is $h_{u}^{0}=u_{1}h_{1}+u_{2}h_{2}.$ This is a linear function, unbounded when $h_{1}^{2}+h_{2}^{2}\neq 0$. Thus, the maximum condition is satisfied if and only if $h_{1}=h_{2}\equiv 0$. The maximized Hamiltonian in this case is $H=\max_{u\in R^{2}}h_{u}^{0}=0.$

Let a,b,c,d∈R be the components of the covector *p* in canonical Darboux coordinates. They change by the law $\dot{p_{i}}=-\frac{\partial h_{u}^{0}}{\partial q_{i}}$.

The Hamiltonian system of PMP has the form

(7) $$\left\{\begin{array}{c} \dot{x}=u_{1}\cos\theta ,\\ \dot{y}=u_{1}\sin\theta ,\\ \dot{\theta }=u_{1}k,\\ \dot{k}=u_{2}, \end{array}\right.\;\left\{\begin{array}{c} \dot{a}=0,\\ \dot{b}=0,\\ \dot{c}=-u_{1}\left(-a\sin\theta +b\cos\theta \right),\\ \dot{d}=-u_{1}\;c. \end{array}\right.$$

The condition $h_{1}=h_{2}\equiv 0$ implies

(8) $$\left\{\begin{array}{c} a\cos\theta +b\sin\theta +ck\equiv 0,\\ d\equiv 0. \end{array}\right.$$

By virtue of system (7), the second identity implies $\dot{d}=-u_{1}\;c\equiv 0$, while differentiation of the first identity implies $u_{2}\;c\equiv 0$. Thus, we have

(9) $$\left(u_{1}^{2}+u_{2}^{2}\right)c^{2}\equiv 0.$$

Since every admissible curve of positive length is a Lipschitz reparameterization of an arclength parameterized admissible one (see ([22], Lemma 3.16)), one can chose the natural parameterization $u_{1}^{2}+u_{2}^{2}=1$ for every trajectory of system (1) that is not a fixed point. Thus, identity (9) is equivalent to c≡0 on the intervals of time where the trajectory is not a fixed point.

The identity c≡0 implies $\dot{c}\equiv 0$. From the third equation of system (7), it follows that $u_{1}\left(-a\sin\theta +b\cos\theta \right)\equiv 0$. On the other hand, the first identity of system (8) takes the form acosθ+bsinθ≡0. Thus, we have $u_{1}^{2}\left(a^{2}+b^{2}\right)\equiv 0$. Note that, if $a^{2}+b^{2}=0$, then a=b=c=d=0, which contradicts the nontriviality condition in PMP. Thus, the case $u_{1}\equiv 0$ remains to be considered.

In this case, the covector *p* is constant and nonzero. We obtain that the abnormal extremals have the form $\gamma \left(t\right)=(0,0,0,k_{0}+U\left(t\right))$, where $U\left(t\right)=\int_{0}^{t}u_{2}\left(\tau \right)d\tau$, and $u_{2}\left(t\right)$ is any real-valued $L^{\infty }\left(0,T\right)$ function. It is easy to see that the trajectory is not optimal if $u_{2}$ changes its sign. This holds since, when $u_{2}$ changes its sign, the trajectory is followed in opposite directions. Choosing the natural parameterization $u_{1}^{2}+u_{2}^{2}=1$ on optimal trajectories, we obtain $u_{2}=\pm 1$. This results in the optimal trajectory $\gamma \left(t\right)=\left(0,0,0,k_{0}\pm t\right)$. Thus, we obtain the following result.

**Theorem** **2.** *The abnormal extremal trajectories in problems* (1)–(3) *have the form $\gamma \left(t\right)=(0,0,0,k_{0}+\int_{0}^{t}u_{2}\left(\tau \right)d\tau )$, where $u_{2}\left(t\right)$ is any real-valued $L^{\infty }\left(0,T\right)$ function. Naturally parameterized abnormal optimal trajectories have the form $\gamma \left(t\right)=\left(0,0,0,k_{0}\pm t\right)$.*

### 4.2. Normal Case ν=−1

The Pontryagin function $h_{u}^{-1}$ takes the form $h_{u}^{-1}=u_{1}h_{1}+u_{2}h_{2}-\left(u_{1}^{2}+u_{2}^{2}\right)/2.$ The maximum condition gives the expression for the extremal controls:

$$\frac{\partial h_{u}^{-1}}{\partial u_{i}}=h_{i}-u_{i}=0\;\Rightarrow \;u_{i}=h_{i}.$$

The maximized Hamiltonian $H=\max_{u\in R^{2}}h_{u}^{-1}$ takes the form

$$H=\frac{h_{1}^{2}+h_{2}^{2}}{2}.$$

By definition $h_{i}=\langle p,X_{i}\rangle$, i=1,…,4 we have

$$\left\{\begin{array}{c} h_{1}=a\cos\theta +b\sin\theta +ck,\\ h_{2}=d,\\ h_{3}=-c,\\ h_{4}=-a\sin\theta +b\cos\theta . \end{array}\right.\;\Leftrightarrow \;\left\{\begin{array}{c} a=\left(h_{1}+kh_{3}\right)\cos\theta -h_{4}\sin\theta ,\\ b=h_{4}\cos\theta +\left(h_{1}+kh_{3}\right)\sin\theta ,\\ c=-h_{3},\\ d=h_{2}. \end{array}\right.$$

The Hamiltonian system in canonical coordinates has the form

$$\left\{\begin{array}{c} \dot{x}=\left(a\cos\theta +b\sin\theta +ck\right)\cos\theta ,\\ \dot{y}=\left(a\cos\theta +b\sin\theta +ck\right)\sin\theta ,\\ \dot{\theta }=k\left(a\cos\theta +b\sin\theta +ck\right),\\ \dot{k}=d, \end{array}\right.\;\left\{\begin{array}{c} \dot{a}=0,\\ \dot{b}=0,\\ \dot{c}=-\left(b\cos\theta -a\sin\theta \right)\left(kc+a\cos\theta +b\sin\theta \right),\\ \dot{d}=-c\left(ck+a\cos\theta +b\sin\theta \right). \end{array}\right.$$

We rewrite this system in the coordinates $h_{i}$:

(10) $$\left\{\begin{array}{c} \dot{x}=h_{1}\cos\theta ,\\ \dot{y}=h_{1}\sin\theta ,\\ \dot{\theta }=h_{1}k,\\ \dot{k}=h_{2}, \end{array}\right.\;\left\{\begin{array}{c} {\dot{h}}_{1}=-h_{2}h_{3},\\ {\dot{h}}_{2}=h_{1}h_{3},\\ {\dot{h}}_{3}=h_{1}h_{4},\\ {\dot{h}}_{4}=-kh_{1}\left(kh_{3}+h_{1}\right). \end{array}\right.$$

By choosing the natural parameterization $u_{1}^{2}+u_{2}^{2}=1$ on the extremal trajectories, one fixes the level surface of the Hamiltonian $H=(h_{1}^{2}+h_{2}^{2})/2=1/2$. A polar angle $\alpha \in S^{1}$ is introduce into the plane $\left(h_{1},h_{2}\right)$:

$$h_{1}=\cos\alpha ,\;h_{2}=\sin\alpha .$$

By rewriting Hamiltonian system (10) we obtain the following result.

**Theorem** **3.** *The naturally parameterized normal extremal trajectories in problems* (1)–(3) *are solutions to the system*

(11) $$\left\{\begin{array}{c} \dot{x}=\cos\alpha \cos\theta ,\\ \dot{y}=\cos\alpha \sin\theta ,\\ \dot{\theta }=k\cos\alpha ,\\ \dot{k}=\sin\alpha , \end{array}\right.\;\left\{\begin{array}{c} \dot{\alpha }=h_{3},\\ {\dot{h}}_{3}=h_{4}\cos\alpha ,\\ {\dot{h}}_{4}=-k\cos\alpha \left(kh_{3}+\cos\alpha \right). \end{array}\right.$$

The question of the existence of an analytic expression for extremal trajectories arises: is the Hamiltonian system integrable? To prove the Liouville integrability of system (10), it suffices to find four functionally independent first integrals in involution. One such integral is the Hamiltonian *H*, and two more first integrals *a* and *b* follow directly from the representation of the Hamiltonian system in canonical coordinates. These three integrals are functionally independent and are in involution. The existence of the remaining first integral is under investigation.

Numerical experiments have been carried out, indicating the presence of the fourth integral. To this end, we consider the four-dimensional system, which is decoupled from the rest of the variables:

(12) $$\left\{\begin{array}{c} \dot{\alpha }=h_{3},\\ {\dot{h}}_{3}=h_{4}\cos\alpha ,\\ {\dot{h}}_{4}=-k\cos\alpha \left(kh_{3}+\cos\alpha \right),\\ \dot{k}=\sin\alpha . \end{array}\right.$$

#### Numerical Simulations

The existence of the first integral of system (12) was studied using the Poincare map. The method consists of three steps. In the first step, one needs to find a periodic trajectory of the system. The second step consists of the construction of a manifold transversal to the flow of the system in a neighborhood of a point of the periodic trajectory. In the third step, small perturbations of the initial point are considered and the corresponding trajectories are computed numerically until they intersect the transversal submanifold several times (*N* times). Such points are called orbits of the Poincare map. If the dynamics exhibit chaotic behavior, the trajectories strongly diverge under a small perturbation of the initial point and the orbits form a set of points chaotically distributed in the transversal submanifold. In the case of an integrable system, an orbit forms a set of points lying in a submanifold of a smaller dimension in the transversal manifold. Next, we perform this method for system (12).

Step 1.

There exists a one-parameter family of periodic trajectories

$$\alpha \left(t\right)=\frac{\pi }{2}+t\;h_{3}\left(0\right),\;\;h_{3}\left(t\right)=h_{3}\left(0\right),\;\;h_{4}\left(t\right)=0,\;\;k\left(t\right)=\frac{\sin(t\;h_{3}\left(0\right))}{h_{3}\left(0\right)},$$

obtained by changing the initial value $h_{3}\left(0\right)$. In this case, the period is $T=|\frac{2\pi }{h_{3}\left(0\right)}|$. With such initial values, the point $\left(h_{3},h_{4}\right)=\left(h_{3}\left(0\right),0\right)$ is fixed. For the next steps, we choose a periodic trajectory passing through the initial point $\alpha \left(0\right)=\frac{\pi }{2}$, $h_{3}\left(0\right)=1$, $h_{4}\left(0\right)=0$, k(0)=0.

Step 2.

It can be checked that the hyperspace k=0 is transversal to the periodic trajectory at the initial point. Indeed, the tangent vector to the periodic trajectory at the initial point is (0,0,0,1), which is orthogonal to the hyperspace k=0.

Step 3.

New initial points were chosen from a small neighborhood of the initial point of the periodic trajectory. The trajectories departing from these points were computed. Points of intersections of every such trajectory with the transversal hyperspace were computed (for the first time, the second time, *…*, the *N*-th time).

**Figure 2:**
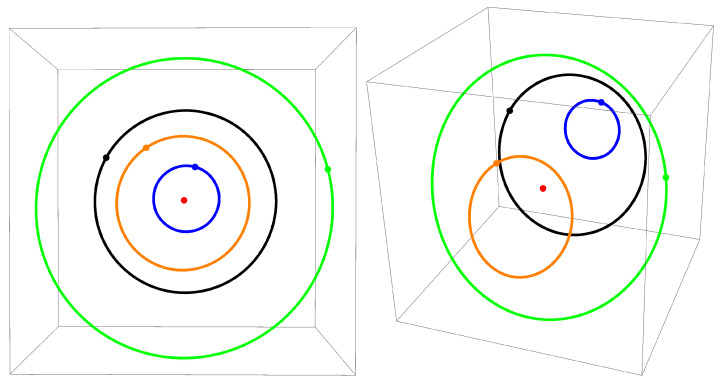
Orbits of the Poincare map in the space $\left(\alpha ,h_{3},h_{4}\right)$ are formed by intersection points of the transversal hyperspace k=0 with trajectories close to the periodic one (red dot). Different orbits are depicted in different colors. Starting points are indicated for each trajectory.

In Figure 2, the orbits of the Poincare map in the space $\left(\alpha ,h_{3},h_{4}\right)$ are shown. The red dot corresponds to the orbit of a periodic trajectory with initial value $\alpha \left(0\right)=\frac{\pi }{2}$, $h_{3}\left(0\right)=1$, $h_{4}\left(0\right)=0$. For the rest of the trajectories, the following parameters were used. For the orange trajectory, α(0)=1.56, $h_{3}\left(0\right)=0.94$, $h_{4}\left(0\right)=0.02$. For the green trajectory, α(0)=1.55, $h_{3}\left(0\right)=1.06$, $h_{4}\left(0\right)=0.05$. For the black trajectory, α(0)=1.6, $h_{3}\left(0\right)=1.14$, $h_{4}\left(0\right)=0.02$. For the blue trajectory, α(0)=1.58, $h_{3}\left(0\right)=1.24$, $h_{4}\left(0\right)=0.01$. The number of iterations of the Poincare map for all trajectories was chosen as N=1000.

The trajectories were computed by numerical integration (NDSolve in Wolfram Mathematica). The instances when the trajectory intersects the transversal hyperspace were determined using the numerical solution (FindRoot) of the equation k(t)=0 along the given trajectory with initial approximation $t=|\frac{2\pi }{h_{3}\left(0\right)}|$ at each iteration i=1,…,N.

It is remarkable that the points of the Poincare map fill continuous closed curves. This indicates that, for a small perturbation of the initial conditions, the trajectories remain close over a long time interval. This situation arises when the system is integrable (at least in some domain). Thus, the presented numerical experiments indicate the presence of a fourth independent first integral, which results in Liouville integrability of the Hamiltonian system (11).

## 5. Conclusions

In this paper, we consider the problem of sub-Riemannian geodesics in the four-dimensional manifold $M=R^{2}\times \mathrm{SO}\left(2\right)\times R$. This problem arises when modelling the mechanism of occluded contours completion in the extended model of the visual cortex by J. Petitot, G. Citti, and A. Sarti. The extension of the classical 3D model is performed by taking the curvature of the contours into account.

The following main results are obtained in the article. Complete controllability of the system and existence of optimal controls are proven. The Hamiltonian system of PMP is derived. The explicit parametrization of abnormal trajectories is found. In the normal case, three functionally independent first integrals are found. A hypothesis is formulated, confirmed by numerical experiments: the normal Hamiltonian system of PMP is Liouville integrable.

Integrability is an important property of the model. This property indicates the adequateness of the model (the visual signal is processed in a deterministic way). It ensures the stability of numerical methods of integration, which appear in brain-inspired image processing algorithms (such as image enchantment, inpainting, and salient curve extraction).

## Data Availability

https://notebookarchive.org//2021-10-1uutypp (accessed on 15 December 2021).

## Abbreviations

The following abbreviations are used in this manuscript:

PMP	Pontryagin maximum principle
